# *N*′-[1-(4-Amino­phen­yl)ethyl]pyrazine-2-carbohydrazide

**DOI:** 10.1107/S1600536810004083

**Published:** 2010-02-06

**Authors:** Zhi-Yong Xing, De-Cheng Yu, Lai-Jun Li, Hai-Yan Liu, Jian-Fei Zhang

**Affiliations:** a College of Pharmacy, Jiamusi University, Jiamusi 154007, People’s Republic of China; b College of Science, Northeast Agricultural University, Harbin 150030, People’s Republic of China; c College of Computer and Information Engineering, Heilongjiang University of Science and Technology, Harbin 150027, People’s Republic of China

## Abstract

The title compound, C_13_H_13_N_5_O, crystallizes with two mol­ecules in the asymmetric unit. The crystal structure is stabilized by intra­molecular N—H⋯N and N—H⋯O hydrogen bonds. The dihedral angles between the pyrazine ring and the 4-aminolphenyl ring are 2.5 (1) and 6.5 (1)° in the two molecules.

## Related literature

For applications of the pyrazine ring system in drug development, see: Du *et al.* (2009 ▶); Dubinina *et al.* (2006 ▶); Ellsworth *et al.* (2007 ▶); Mukaiyama *et al.* (2007 ▶).
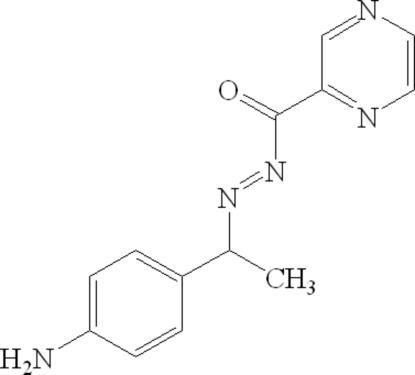


## Experimental

### 

#### Crystal data

C_13_H_13_N_5_O
*M* *_r_* = 255.28
Triclinic, 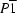

*a* = 6.9783 (13) Å
*b* = 10.689 (3) Å
*c* = 17.061 (5) Å
α = 106.971 (10)°
β = 98.499 (4)°
γ = 90.174 (14)°
*V* = 1202.3 (5) Å^3^
*Z* = 4
Mo *K*α radiation
μ = 0.10 mm^−1^
*T* = 113 K
0.10 × 0.09 × 0.04 mm

#### Data collection

Rigaku Saturn diffractometer
Absorption correction: multi-scan (*REQAB*; Jacobson, 1998 ▶) *T*_min_ = 0.991, *T*_max_ = 0.996
9090 measured reflections
4150 independent reflections
2832 reflections with *I* > 2σ(*I*)
*R*_int_ = 0.052

#### Refinement

*R*[*F* ^2^ > 2σ(*F* ^2^)] = 0.069
*wR*(*F* ^2^) = 0.164
*S* = 1.05
4150 reflections
364 parameters
H atoms treated by a mixture of independent and constrained refinement
Δρ_max_ = 0.26 e Å^−3^
Δρ_min_ = −0.30 e Å^−3^

### 

Data collection: *CrystalClear* (Rigaku/MSC, 2005 ▶); cell refinement: *CrystalClear*; data reduction: *CrystalStructure* (Rigaku/MSC, 2005 ▶); program(s) used to solve structure: *SHELXS97* (Sheldrick, 2008 ▶); program(s) used to refine structure: *SHELXL97* (Sheldrick, 2008 ▶); molecular graphics: *SHELXTL* (Sheldrick, 2008 ▶); software used to prepare material for publication: *SHELXTL*.

## Supplementary Material

Crystal structure: contains datablocks global, I. DOI: 10.1107/S1600536810004083/hg2638sup1.cif

Structure factors: contains datablocks I. DOI: 10.1107/S1600536810004083/hg2638Isup2.hkl

Additional supplementary materials:  crystallographic information; 3D view; checkCIF report

## Figures and Tables

**Table 1:** Hydrogen-bond geometry (Å, °)

*D*—H⋯*A*	*D*—H	H⋯*A*	*D*⋯*A*	*D*—H⋯*A*
N1—H1*A*⋯N10^i^	0.88 (3)	2.16 (3)	3.030 (4)	170 (3)
N1—H1*B*⋯O2^ii^	0.97 (3)	1.89 (3)	2.852 (3)	171 (3)
N6—H6*A*⋯N1^iii^	0.91 (3)	2.38 (3)	3.162 (4)	145 (3)
N6—H6*B*⋯O1^ii^	0.95 (3)	2.07 (3)	3.015 (3)	169 (3)

Symmetry codes: (i) 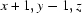; (ii) 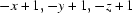; (iii) 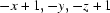.

**Fig. 1.**
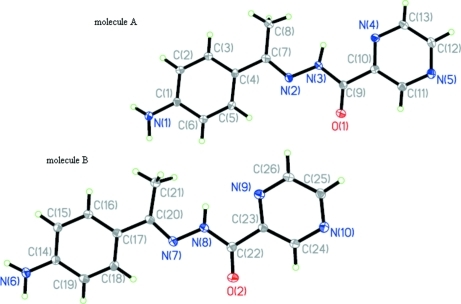
The structure of (I), showing the atom-labelling scheme. Displacement ellipsoids are drawn at the 30% probability level.

**Fig. 2.**
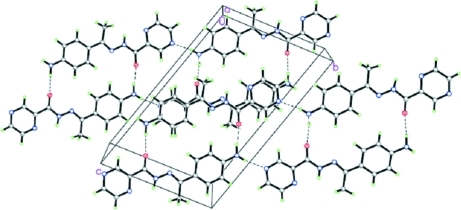
The molecular packing depicting N—H···O and N—H···N intermolecular interactions as dashed lines.

## supplementary crystallographic information

### Comment

The pyrazine ring system is a useful structural element in medicinal chemistry and has found broad applications in drug development which can be used as antiproliferative agent (Dubinina *et al.*, 2006), potent CXCR3 antagonists (Du *et al.*, 2009), CB1 antagonists (Ellsworth *et al.*, 2007) and c-Src inhibitory (Mukaiyama *et al.*, 2007). In view of different applications of this class of compounds, we have undertaken a single-crystal structure determination of the title compound. The crystal structure has two independent molecules in the aysmmetric unit, and the dihedral angles between the pyrazine ring and the 4-aminolphenyl ring are 2.5 (1) and 6.5 (1)° in the two molecules (Fig. 1). The crystal structure is stabilized by N—H···O intermolecular hydrogen bonds (between molecules of the 'A' type), each of which are also by N—H···N intermolecular interactions (with molecules of 'B' type) between them (Fig. 2).

### Experimental

For the synthesis of *N*'-(1-(4-aminophenyl)ethylidene) pyrazine-2-carbohydrazide, (I), a mixture of pyrazine-2-carboxylic acid hydrazide (0.01 mol, 1.38 g) and 1-(4-aminophenyl)ethanone (0.01 mol, 1.35 g) in methanol was refluxed for 2 h. The solid material obtained on cooling was filtered, washed with ethanol: ether =1:1, dried and crystallized from methanol (yield 62%). The compound (1.0 mmol, 0.268 g) was dissolved in 95% ethanol (30 ml) and kept at room temperature for one week, after which yellow platelet shaped single crystals formed and were collected and washed with ether for X-ray diffraction analysis.

### Refinement

All H atoms were initially located in a difference Fourier map. The C—Hatoms were then constrained to an ideal geometry, with C(CH_3_)—H distances of 0.98 Å, *C*(phenyl)—H distances of 0.93 Å and U_iso_(H) = 1.2U_eq_(C). The amino H atoms were refined freely with N—H distances in the range 0.88–0.97 Å.

### Crystal data

**Table:**

C_13_H_13_N_5_O	*Z* = 4
*M**_r_* = 255.28	*F*(000) = 536
Triclinic, *P*1	*D*_x_ = 1.410 Mg m^−^^3^
*a* = 6.9783 (13) Å	Mo *K*α radiation, λ = 0.71070 Å
*b* = 10.689 (3) Å	Cell parameters from 2002 reflections
*c* = 17.061 (5) Å	θ = 2.5–27.8°
α = 106.971 (10)°	µ = 0.10 mm^−^^1^
β = 98.499 (4)°	*T* = 113 K
γ = 90.174 (14)°	Platelet, yellow
*V* = 1202.3 (5) Å^3^	0.10 × 0.09 × 0.04 mm

### Data collection

**Table:**

Rigaku Saturn diffractometer	4150 independent reflections
Radiation source: rotating anode	2832 reflections with *I* > 2σ(*I*)
confocal	*R*_int_ = 0.052
Detector resolution: 7.31 pixels mm^-1^	θ_max_ = 25.0°, θ_min_ = 2.0°
ω scans	*h* = −8→8
Absorption correction: multi-scan (REQAB; Jacobson, 1998)	*k* = −11→12
*T*_min_ = 0.991, *T*_max_ = 0.996	*l* = −20→20
9090 measured reflections	

### Refinement

**Table:**

Refinement on *F*^2^	Primary atom site location: structure-invariant direct methods
Least-squares matrix: full	Secondary atom site location: difference Fourier map
*R*[*F*^2^ > 2σ(*F*^2^)] = 0.069	Hydrogen site location: inferred from neighbouring sites
*wR*(*F*^2^) = 0.164	H atoms treated by a mixture of independent and constrained refinement
*S* = 1.05	*w* = 1/[σ^2^(*F*_o_^2^) + (0.0724*P*)^2^] where *P* = (*F*_o_^2^ + 2*F*_c_^2^)/3
4150 reflections	(Δ/σ)_max_ = 0.001
364 parameters	Δρ_max_ = 0.26 e Å^−^^3^
0 restraints	Δρ_min_ = −0.29 e Å^−^^3^

### Special details

**Table:**

Geometry. All esds (except the esd in the dihedral angle between two l.s. planes) are estimated using the full covariance matrix. The cell esds are taken into account individually in the estimation of esds in distances, angles and torsion angles; correlations between esds in cell parameters are only used when they are defined by crystal symmetry. An approximate (isotropic) treatment of cell esds is used for estimating esds involving l.s. planes.
Refinement. Refinement of *F*^2^ against ALL reflections. The weighted *R*-factor wR and goodness of fit *S* are based on *F*^2^, conventional *R*-factors *R* are based on *F*, with *F* set to zero for negative *F*^2^. The threshold expression of *F*^2^ > σ(*F*^2^) is used only for calculating *R*-factors(gt) etc. and is not relevant to the choice of reflections for refinement. *R*-factors based on *F*^2^ are statistically about twice as large as those based on *F*, and *R*- factors based on ALL data will be even larger.

### Fractional atomic coordinates and isotropic or equivalent isotropic displacement parameters (Å^2^)

**Table:**

	*x*	*y*	*z*	*U*_iso_*/*U*_eq_	
C1	0.8650 (4)	0.1637 (3)	0.24329 (16)	0.0213 (6)	
C2	0.8881 (4)	0.0891 (3)	0.16283 (16)	0.0243 (7)	
H2	0.9195	−0.0001	0.1527	0.029*	
C3	0.8657 (4)	0.1438 (3)	0.09828 (17)	0.0220 (6)	
H3	0.8823	0.0913	0.0445	0.026*	
C4	0.8193 (4)	0.2747 (3)	0.11007 (16)	0.0197 (6)	
C5	0.7963 (4)	0.3486 (3)	0.19089 (16)	0.0220 (6)	
H5	0.7631	0.4375	0.2009	0.026*	
C6	0.8207 (4)	0.2952 (3)	0.25550 (17)	0.0239 (7)	
H6	0.8074	0.3483	0.3096	0.029*	
C7	0.7939 (4)	0.3348 (3)	0.04141 (16)	0.0217 (6)	
C8	0.8024 (4)	0.2514 (3)	−0.04611 (16)	0.0289 (7)	
H8A	0.7298	0.2917	−0.0854	0.043*	
H8B	0.7451	0.1638	−0.0546	0.043*	
H8C	0.9379	0.2447	−0.0552	0.043*	
C9	0.6961 (4)	0.6487 (3)	0.02186 (16)	0.0217 (6)	
C10	0.6788 (4)	0.7066 (3)	−0.04848 (16)	0.0203 (6)	
C11	0.6397 (4)	0.8372 (3)	−0.03596 (17)	0.0238 (7)	
H11	0.6214	0.8884	0.0179	0.029*	
C12	0.6529 (4)	0.8158 (3)	−0.17048 (17)	0.0268 (7)	
H12	0.6445	0.8509	−0.2159	0.032*	
C13	0.6926 (4)	0.6840 (3)	−0.18385 (17)	0.0256 (7)	
H13	0.7104	0.6328	−0.2378	0.031*	
C14	0.4201 (4)	0.1709 (3)	0.68980 (17)	0.0227 (6)	
C15	0.3869 (4)	0.0943 (3)	0.60680 (17)	0.0256 (7)	
H15	0.3857	0.0016	0.5936	0.031*	
C16	0.3557 (4)	0.1525 (3)	0.54366 (17)	0.0249 (7)	
H16	0.3359	0.0986	0.4877	0.030*	
C17	0.3526 (4)	0.2884 (3)	0.56009 (16)	0.0221 (6)	
C18	0.3846 (4)	0.3637 (3)	0.64354 (17)	0.0246 (7)	
H18	0.3836	0.4564	0.6569	0.029*	
C19	0.4175 (4)	0.3068 (3)	0.70710 (17)	0.0250 (7)	
H19	0.4386	0.3606	0.7631	0.030*	
C20	0.3116 (4)	0.3502 (3)	0.49280 (16)	0.0222 (6)	
C21	0.3380 (4)	0.2769 (3)	0.40568 (17)	0.0299 (7)	
H21A	0.2126	0.2644	0.3692	0.045*	
H21B	0.3896	0.1913	0.4046	0.045*	
H21C	0.4289	0.3271	0.3865	0.045*	
C22	0.1804 (4)	0.6521 (3)	0.46356 (17)	0.0242 (7)	
C23	0.1331 (4)	0.6910 (3)	0.38597 (16)	0.0214 (6)	
C24	0.1030 (4)	0.8204 (3)	0.38957 (17)	0.0261 (7)	
H24	0.1126	0.8839	0.4424	0.031*	
C25	0.0467 (4)	0.7651 (3)	0.24967 (17)	0.0262 (7)	
H25	0.0152	0.7878	0.1996	0.031*	
C26	0.0763 (4)	0.6349 (3)	0.24498 (17)	0.0265 (7)	
H26	0.0644	0.5714	0.1921	0.032*	
N1	0.8761 (4)	0.1101 (2)	0.30779 (15)	0.0294 (6)	
H1A	0.933 (5)	0.036 (3)	0.3056 (18)	0.044*	
H1B	0.861 (4)	0.171 (3)	0.361 (2)	0.044*	
N2	0.7625 (3)	0.4591 (2)	0.06300 (13)	0.0228 (5)	
N3	0.7388 (3)	0.5209 (2)	0.00192 (14)	0.0224 (6)	
H3A	0.742 (4)	0.482 (3)	−0.0522 (17)	0.027*	
N4	0.7064 (3)	0.6282 (2)	−0.12325 (13)	0.0228 (5)	
N5	0.6263 (3)	0.8945 (2)	−0.09690 (14)	0.0264 (6)	
N6	0.4577 (4)	0.1131 (3)	0.75362 (16)	0.0289 (6)	
H6A	0.410 (5)	0.029 (3)	0.7404 (18)	0.043*	
H6B	0.433 (4)	0.168 (3)	0.8058 (19)	0.043*	
N7	0.2571 (3)	0.4699 (2)	0.51400 (14)	0.0257 (6)	
N8	0.2176 (3)	0.5256 (2)	0.44979 (14)	0.0238 (6)	
H8	0.198 (4)	0.475 (3)	0.3982 (17)	0.029*	
N9	0.1215 (3)	0.5968 (2)	0.31366 (14)	0.0257 (6)	
N10	0.0608 (3)	0.8597 (2)	0.32169 (14)	0.0277 (6)	
O1	0.6759 (3)	0.71548 (19)	0.09187 (12)	0.0328 (5)	
O2	0.1874 (3)	0.73296 (19)	0.53239 (11)	0.0347 (5)	

### Atomic displacement parameters (Å^2^)

**Table:**

	*U*^11^	*U*^22^	*U*^33^	*U*^12^	*U*^13^	*U*^23^
C1	0.0209 (14)	0.0198 (15)	0.0229 (16)	−0.0025 (11)	−0.0002 (11)	0.0074 (12)
C2	0.0294 (15)	0.0212 (16)	0.0211 (16)	0.0025 (12)	0.0050 (12)	0.0038 (12)
C3	0.0214 (14)	0.0227 (16)	0.0206 (15)	0.0012 (12)	0.0036 (11)	0.0044 (12)
C4	0.0185 (13)	0.0181 (15)	0.0241 (16)	−0.0013 (11)	0.0033 (11)	0.0086 (12)
C5	0.0218 (14)	0.0180 (15)	0.0263 (16)	0.0008 (11)	0.0031 (12)	0.0069 (12)
C6	0.0284 (15)	0.0227 (16)	0.0204 (15)	0.0025 (12)	0.0053 (12)	0.0050 (12)
C7	0.0167 (13)	0.0245 (16)	0.0220 (15)	−0.0024 (11)	0.0026 (11)	0.0042 (12)
C8	0.0337 (16)	0.0316 (18)	0.0224 (16)	0.0013 (13)	0.0044 (13)	0.0092 (13)
C9	0.0239 (14)	0.0208 (16)	0.0204 (16)	−0.0003 (12)	0.0022 (12)	0.0066 (12)
C10	0.0170 (13)	0.0212 (15)	0.0221 (15)	0.0008 (11)	0.0043 (11)	0.0048 (12)
C11	0.0236 (15)	0.0249 (16)	0.0221 (16)	−0.0021 (12)	0.0019 (12)	0.0064 (13)
C12	0.0265 (15)	0.0317 (18)	0.0258 (17)	0.0006 (13)	0.0026 (12)	0.0147 (14)
C13	0.0255 (15)	0.0332 (18)	0.0192 (15)	0.0011 (13)	0.0047 (12)	0.0088 (13)
C14	0.0199 (14)	0.0238 (16)	0.0262 (16)	0.0032 (12)	0.0066 (12)	0.0087 (13)
C15	0.0293 (16)	0.0211 (16)	0.0287 (17)	0.0026 (12)	0.0099 (13)	0.0082 (13)
C16	0.0292 (15)	0.0238 (16)	0.0215 (16)	0.0011 (12)	0.0067 (12)	0.0050 (12)
C17	0.0195 (14)	0.0248 (16)	0.0219 (15)	0.0026 (12)	0.0046 (11)	0.0063 (12)
C18	0.0248 (15)	0.0216 (16)	0.0267 (16)	0.0031 (12)	0.0020 (12)	0.0071 (13)
C19	0.0222 (14)	0.0301 (17)	0.0205 (15)	−0.0007 (12)	0.0022 (12)	0.0043 (13)
C20	0.0194 (14)	0.0277 (17)	0.0197 (15)	−0.0007 (12)	0.0040 (11)	0.0069 (13)
C21	0.0376 (17)	0.0279 (17)	0.0244 (17)	0.0056 (13)	0.0060 (13)	0.0074 (13)
C22	0.0219 (14)	0.0234 (16)	0.0265 (17)	−0.0014 (12)	0.0035 (12)	0.0061 (13)
C23	0.0207 (14)	0.0235 (16)	0.0198 (15)	−0.0002 (12)	0.0049 (11)	0.0053 (12)
C24	0.0303 (16)	0.0268 (17)	0.0211 (16)	0.0037 (13)	0.0058 (12)	0.0060 (13)
C25	0.0258 (15)	0.0317 (18)	0.0235 (16)	0.0078 (13)	0.0043 (12)	0.0113 (14)
C26	0.0269 (15)	0.0300 (17)	0.0203 (16)	0.0018 (13)	0.0050 (12)	0.0036 (13)
N1	0.0450 (16)	0.0207 (15)	0.0250 (14)	0.0080 (12)	0.0097 (12)	0.0087 (12)
N2	0.0246 (13)	0.0237 (14)	0.0217 (13)	0.0013 (10)	0.0013 (10)	0.0103 (11)
N3	0.0290 (13)	0.0224 (14)	0.0166 (13)	0.0012 (10)	0.0036 (10)	0.0073 (11)
N4	0.0238 (12)	0.0244 (13)	0.0203 (13)	0.0017 (10)	0.0044 (10)	0.0064 (10)
N5	0.0277 (13)	0.0249 (14)	0.0292 (14)	−0.0004 (10)	0.0057 (10)	0.0116 (11)
N6	0.0358 (15)	0.0285 (15)	0.0267 (15)	0.0041 (12)	0.0074 (11)	0.0135 (12)
N7	0.0305 (13)	0.0272 (14)	0.0224 (13)	0.0024 (11)	0.0057 (10)	0.0110 (11)
N8	0.0300 (13)	0.0231 (14)	0.0176 (13)	0.0013 (10)	0.0028 (10)	0.0055 (11)
N9	0.0250 (12)	0.0284 (14)	0.0227 (13)	0.0024 (10)	0.0033 (10)	0.0062 (11)
N10	0.0305 (13)	0.0252 (14)	0.0289 (14)	0.0031 (11)	0.0058 (11)	0.0096 (11)
O1	0.0459 (13)	0.0313 (12)	0.0214 (12)	0.0075 (10)	0.0080 (9)	0.0068 (9)
O2	0.0509 (14)	0.0298 (12)	0.0199 (12)	0.0042 (10)	0.0042 (9)	0.0027 (9)

### Geometric parameters (Å, °)

**Table:**

C1—N1	1.374 (3)	C15—H15	0.9500
C1—C6	1.402 (4)	C16—C17	1.398 (4)
C1—C2	1.405 (3)	C16—H16	0.9500
C2—C3	1.380 (4)	C17—C18	1.400 (4)
C2—H2	0.9500	C17—C20	1.477 (4)
C3—C4	1.400 (3)	C18—C19	1.383 (4)
C3—H3	0.9500	C18—H18	0.9500
C4—C5	1.408 (4)	C19—H19	0.9500
C4—C7	1.481 (4)	C20—N7	1.298 (3)
C5—C6	1.372 (4)	C20—C21	1.503 (4)
C5—H5	0.9500	C21—H21A	0.9800
C6—H6	0.9500	C21—H21B	0.9800
C7—N2	1.300 (3)	C21—H21C	0.9800
C7—C8	1.509 (4)	C22—O2	1.232 (3)
C8—H8A	0.9800	C22—N8	1.336 (3)
C8—H8B	0.9800	C22—C23	1.493 (4)
C8—H8C	0.9800	C23—N9	1.337 (3)
C9—O1	1.228 (3)	C23—C24	1.385 (4)
C9—N3	1.353 (3)	C24—N10	1.337 (3)
C9—C10	1.493 (4)	C24—H24	0.9500
C10—N4	1.348 (3)	C25—N10	1.334 (3)
C10—C11	1.383 (4)	C25—C26	1.388 (4)
C11—N5	1.345 (3)	C25—H25	0.9500
C11—H11	0.9500	C26—N9	1.345 (3)
C12—N5	1.331 (3)	C26—H26	0.9500
C12—C13	1.395 (4)	N1—H1A	0.88 (3)
C12—H12	0.9500	N1—H1B	0.97 (3)
C13—N4	1.329 (3)	N2—N3	1.379 (3)
C13—H13	0.9500	N3—H3A	0.90 (3)
C14—N6	1.394 (4)	N6—H6A	0.91 (3)
C14—C19	1.396 (4)	N6—H6B	0.95 (3)
C14—C15	1.398 (4)	N7—N8	1.387 (3)
C15—C16	1.384 (4)	N8—H8	0.88 (3)
			
N1—C1—C6	120.2 (2)	C16—C17—C18	117.0 (2)
N1—C1—C2	122.1 (3)	C16—C17—C20	121.7 (2)
C6—C1—C2	117.6 (2)	C18—C17—C20	121.3 (3)
C3—C2—C1	120.8 (3)	C19—C18—C17	121.8 (3)
C3—C2—H2	119.6	C19—C18—H18	119.1
C1—C2—H2	119.6	C17—C18—H18	119.1
C2—C3—C4	121.7 (2)	C18—C19—C14	120.6 (3)
C2—C3—H3	119.1	C18—C19—H19	119.7
C4—C3—H3	119.1	C14—C19—H19	119.7
C3—C4—C5	117.1 (2)	N7—C20—C17	116.3 (2)
C3—C4—C7	122.4 (2)	N7—C20—C21	123.2 (2)
C5—C4—C7	120.5 (2)	C17—C20—C21	120.5 (2)
C6—C5—C4	121.4 (3)	C20—C21—H21A	109.5
C6—C5—H5	119.3	C20—C21—H21B	109.5
C4—C5—H5	119.3	H21A—C21—H21B	109.5
C5—C6—C1	121.3 (3)	C20—C21—H21C	109.5
C5—C6—H6	119.3	H21A—C21—H21C	109.5
C1—C6—H6	119.3	H21B—C21—H21C	109.5
N2—C7—C4	115.2 (2)	O2—C22—N8	125.4 (3)
N2—C7—C8	125.0 (2)	O2—C22—C23	121.2 (3)
C4—C7—C8	119.8 (2)	N8—C22—C23	113.5 (2)
C7—C8—H8A	109.5	N9—C23—C24	121.7 (2)
C7—C8—H8B	109.5	N9—C23—C22	117.6 (2)
H8A—C8—H8B	109.5	C24—C23—C22	120.7 (2)
C7—C8—H8C	109.5	N10—C24—C23	122.7 (3)
H8A—C8—H8C	109.5	N10—C24—H24	118.7
H8B—C8—H8C	109.5	C23—C24—H24	118.7
O1—C9—N3	124.3 (3)	N10—C25—C26	122.6 (3)
O1—C9—C10	121.0 (2)	N10—C25—H25	118.7
N3—C9—C10	114.7 (2)	C26—C25—H25	118.7
N4—C10—C11	121.8 (2)	N9—C26—C25	121.5 (2)
N4—C10—C9	117.7 (2)	N9—C26—H26	119.3
C11—C10—C9	120.5 (2)	C25—C26—H26	119.3
N5—C11—C10	122.7 (3)	C1—N1—H1A	121 (2)
N5—C11—H11	118.6	C1—N1—H1B	114.9 (18)
C10—C11—H11	118.6	H1A—N1—H1B	120 (3)
N5—C12—C13	122.9 (3)	C7—N2—N3	117.9 (2)
N5—C12—H12	118.5	C9—N3—N2	119.2 (2)
C13—C12—H12	118.5	C9—N3—H3A	115.6 (17)
N4—C13—C12	121.9 (3)	N2—N3—H3A	125.0 (17)
N4—C13—H13	119.1	C13—N4—C10	115.8 (2)
C12—C13—H13	119.1	C12—N5—C11	114.9 (2)
N6—C14—C19	120.8 (3)	C14—N6—H6A	114.6 (19)
N6—C14—C15	120.9 (3)	C14—N6—H6B	113.5 (18)
C19—C14—C15	118.3 (2)	H6A—N6—H6B	114 (3)
C16—C15—C14	120.6 (3)	C20—N7—N8	115.4 (2)
C16—C15—H15	119.7	C22—N8—N7	121.9 (2)
C14—C15—H15	119.7	C22—N8—H8	117.4 (18)
C15—C16—C17	121.7 (3)	N7—N8—H8	119.9 (18)
C15—C16—H16	119.1	C23—N9—C26	116.1 (2)
C17—C16—H16	119.1	C25—N10—C24	115.4 (2)
			
N1—C1—C2—C3	176.4 (2)	C16—C17—C20—N7	160.7 (2)
C6—C1—C2—C3	−0.7 (4)	C18—C17—C20—N7	−17.4 (4)
C1—C2—C3—C4	−0.1 (4)	C16—C17—C20—C21	−20.8 (4)
C2—C3—C4—C5	0.1 (4)	C18—C17—C20—C21	161.1 (3)
C2—C3—C4—C7	−179.7 (2)	O2—C22—C23—N9	−177.7 (2)
C3—C4—C5—C6	0.8 (4)	N8—C22—C23—N9	4.0 (3)
C7—C4—C5—C6	−179.4 (2)	O2—C22—C23—C24	2.5 (4)
C4—C5—C6—C1	−1.6 (4)	N8—C22—C23—C24	−175.9 (2)
N1—C1—C6—C5	−175.7 (2)	N9—C23—C24—N10	0.0 (4)
C2—C1—C6—C5	1.5 (4)	C22—C23—C24—N10	179.8 (2)
C3—C4—C7—N2	−176.1 (2)	N10—C25—C26—N9	−0.1 (4)
C5—C4—C7—N2	4.2 (3)	C4—C7—N2—N3	179.6 (2)
C3—C4—C7—C8	4.9 (4)	C8—C7—N2—N3	−1.4 (4)
C5—C4—C7—C8	−174.9 (2)	O1—C9—N3—N2	−0.1 (4)
O1—C9—C10—N4	178.8 (2)	C10—C9—N3—N2	178.5 (2)
N3—C9—C10—N4	0.1 (3)	C7—N2—N3—C9	176.7 (2)
O1—C9—C10—C11	0.0 (4)	C12—C13—N4—C10	0.2 (4)
N3—C9—C10—C11	−178.6 (2)	C11—C10—N4—C13	−0.3 (4)
N4—C10—C11—N5	0.0 (4)	C9—C10—N4—C13	−179.0 (2)
C9—C10—C11—N5	178.7 (2)	C13—C12—N5—C11	−0.3 (4)
N5—C12—C13—N4	0.1 (4)	C10—C11—N5—C12	0.3 (4)
N6—C14—C15—C16	−178.0 (2)	C17—C20—N7—N8	−179.4 (2)
C19—C14—C15—C16	1.1 (4)	C21—C20—N7—N8	2.1 (4)
C14—C15—C16—C17	−1.2 (4)	O2—C22—N8—N7	4.3 (4)
C15—C16—C17—C18	0.7 (4)	C23—C22—N8—N7	−177.4 (2)
C15—C16—C17—C20	−177.5 (2)	C20—N7—N8—C22	−172.7 (2)
C16—C17—C18—C19	−0.1 (4)	C24—C23—N9—C26	−0.9 (4)
C20—C17—C18—C19	178.1 (2)	C22—C23—N9—C26	179.2 (2)
C17—C18—C19—C14	0.1 (4)	C25—C26—N9—C23	1.0 (4)
N6—C14—C19—C18	178.5 (2)	C26—C25—N10—C24	−0.9 (4)
C15—C14—C19—C18	−0.6 (4)	C23—C24—N10—C25	0.9 (4)

### Hydrogen-bond geometry (Å, °)

**Table:**

*D*—H···*A*	*D*—H	H···*A*	*D*···*A*	*D*—H···*A*
N1—H1A···N10^i^	0.88 (3)	2.16 (3)	3.030 (4)	170 (3)
N1—H1B···O2^ii^	0.97 (3)	1.89 (3)	2.852 (3)	171 (3)
N6—H6A···N1^iii^	0.91 (3)	2.38 (3)	3.162 (4)	145 (3)
N6—H6B···O1^ii^	0.95 (3)	2.07 (3)	3.015 (3)	169 (3)

Symmetry codes: (i) *x*+1, *y*−1, *z*; (ii) −*x*+1, −*y*+1, −*z*+1; (iii) −*x*+1, −*y*, −*z*+1.
